# Lung resistance-related protein (LRP) predicts favorable therapeutic outcome in Acute Myeloid Leukemia

**DOI:** 10.1038/s41598-018-36780-8

**Published:** 2019-01-23

**Authors:** Bibi Kulsoom, Tahir Sultan Shamsi, Nasir Ali Afsar

**Affiliations:** 1 National Institute of Blood Diseases and Bone Marrow Transplantation, Karachi, Pakistan; 2 Jinnah Medical and Dental College, Karachi, Pakistan

## Abstract

There is conflicting evidence that MDR1, MRP2 and LRP expression is responsible for chemotherapy resistance. We conducted this study to explore their role in AML therapy outcomes. Bone marrow and peripheral blood samples of 90 AML patients, receiving chemotherapy, were analyzed by real time PCR. Gene expression was calculated by the 2^−ΔΔCt^ method. The patients who had a persistent remission were labelled ‘Good Responder’ (GRes) whereas, those with relapse or drug resistance were labelled ‘Poor Responders’ (PRes). Higher LRP expression in bone marrow, but not in peripheral blood, was positively associated with persistent remission (p = 0.001), GRes (p = 0.002), 1-year overall as well as disease-free survival (p = 0.02 and p = 0.007, respectively). Marrow and blood MDR1 and MRP2 expression did not differ significantly between the above groups. Logistic regression analysis showed that only a diagnosis of acute promyelocytic leukemia (APL; M3) or high marrow LRP expression significantly predicted a favorable therapeutic outcome. This is the first report showing that high bone marrow LRP expression predicts significant favorable therapeutic outcome. Peripheral blood LRP expression as well as marrow and blood MDR1 and MRP2 expression have no predictive value in AML patients treated with standard dose cytarabine and daunorubicin 3+7 regimen.

## Introduction

Successful chemotherapeutic treatment in acute myeloid leukemia (AML) remains a challenge as a substantial number of patients do not achieve complete remission (CR) and many of those who do respond relapse later^1–3^. Although drug resistance has remained a point of focus for many researchers, a lot more still needs to be explored. Since the presence of a drug inside target cells is imperative for successful treatment, the role of efflux transporters, such as ATP-binding cassette (ABC) transporters, is also implicated^4^.

One of the ABC transporter family member, ABCB1, also called multidrug resistance protein 1 (MDR1) or permeability-glycoprotein (P-gp), is involved in cellular efflux of xenobiotics, including chemotherapeutic agents. Researchers have focused on MDR1 expression in many drug resistant hematological and solid cancers, yielding inconsistent results^5–10^.

Another ABC transporter, ABCC2, also called multidrug resistance-associated protein 2 (MRP2), (formerly known as canalicular multispecific organic anion transporter - cMOAT) is commonly found on hepatocyte canaliculi, intestines and kidney cells, and transports various chemicals including drugs^11^. Like MDR1, overexpression of MRP2 has also been related to chemo-resistance^12,13^.

A third protein is lung resistance-related protein (LRP), also known as major vault protein (MVP or VAULT1). LRP is described as a drug efflux transporter and has been accredited to impart chemo-resistance. Although the function of LRP is still not fully understood, its role in the formation of barrel-shaped vault organelles is recognized. Vaults transport different molecules between nucleus and cytoplasm. In addition to MVP, vaults contain vault poly-ADP-ribose polymerase (vPARP), telomerase-associated protein 1 (TEP1) and vault RNA (vRNA). vPARP identifies DNA damage and adds PAR so that the DNA damage is tagged for repair, while TEP1 is involved in telomere formation^14^. LRP is normally expressed in bone marrow^15^. Positive or higher expression has been associated with adverse outcomes in leukemia^9,10^ as well as multiple solid tumors^16,17^. In this study we explored the association of gene expression of MDR1, MRP2 and LRP with clinical outcomes of AML chemotherapy.

## Results

Baseline characteristics are given in Table 1. Most of the patients were between 15–40 years, and the most predominant type was “AML with maturation” (48.9%). Myeloperoxidase (MPO) was tested to establish myeloid linage in 76 patients, of which 62 were positive. Patient data for FLT3, NPM1, PML-RARα, MLL mutation and karyotyping was available only for a limited number of patients (Table 1). 56 patients (62%) achieved CR after first induction, however 19 (34% of CR; 21% of total) relapsed later. Resistant and relapsed patients were collectively labelled as ‘poor responders’ (PRes) (58.9%), while patients with persistent remission (41.1%) were labeled ‘good responders’ (GRes).

**Table 1:** Baseline Characteristics of the Study Population (AML patients, N = 90).

Parameters		N	Percent
Age groups	<15 Years	3	3.3
	15–40 Years	62	68.9
	41–60 Years	24	26.7
	>60 Years	1	1.1
Gender	Male	66	73.3
	Female	24	26.7
AML classification (who)	APL (M3) with t 15:17	17	18.9
	AML without maturation (M1)	15	16.7
	AML with maturation (M2)	44	48.9
	Others:	8	8.9
	-Translocation 6:9	2	2.2
	-AML with minimal differentiation (M0)	2	2.2
	-Acute Myelomonocytic Leukemia (M4)	2	2.2
	-Acute Panmyelosis with fibrosis	1	1.1
	-Myeloid proliferations related to Down syndrome	1	1.1
	Unknown	6	6.7
MPO status	Negative	14	15.6
	Positive	62	68.9
	Unknown	14	15.6
FLT3 mutation	Negative	35	38.9
	Positive	7	7.8
	Unknown	48	53.3
NPM1 mutation	Negative	13	14.4
	Unknown	77	85.6
PML-RAR mutation	Negative	4	4.4
	Positive	5	5.6
	Unknown	81	90.0
MLL mutation	Negative	10	11.1
	Positive	5	5.6
	Unknown	75	83.3
Karyotyping	Unfavorable	18	20.0
	Favorable (APL)	7	7.8
	Normal	24	26.7
	Unknown	41	45.6
Therapeutic response	Resistant	34	37.8
	Relapse	19	21.1
	Persistant Remission	37	41.1
Survival status	Died	42	46.7
	Alive	44	48.9
	Unknown	4	4.4
Final outcome	Poor (Resistant + Relapse)	53	58.9
	Good (Persistent Remission)	37	41.1

Medians and interquartile ranges (IQRs) for MDR1, MRP2 and LRP gene expression are given in Table 2, and boxplots using a logarithmic scale are given in Supplementary Fig. 1. Overall, LRP expression was much higher than MDR1 and MRP2. Median bone marrow LRP expression was higher in subgroups with a better clinical outcome, i.e. APL, negative MPO, persistent remission and being alive. However, peripheral blood LRP expression only partially followed this trend. Median MDR1 and MRP2 expression in bone marrow as well as in peripheral blood were comparable. The Cq value boxplots (linear scale) of the house-keeping gene GAPDH are also given for comparison and as an indicator of quality control.

**Table 2:** Median expression values (and inter-quartile ranges) of MDR-1, MRP-2 and LRP among study population.

Parameters	Bone Marrow										Blood									
	*N*	MDR-1			MRP-2			LRP			*N*	MDR-1			MRP-2			LRP		
		Med	25th	75th	Med	25th	75th	Med	25th	75th		Med	25th	75th	Med	25th	75th	Med	25th	75th
**AML Classification**																				
APL (M3); t15:17	17	0.06	0.01	0.11	0.15	0.00	0.68	3.23	0.34	15.70	14	0.06	0.03	0.20	0.02	0.00	0.13	0.71	0.29	3.94
AML without maturation (M1)	14	0.03	0.00	0.35	0.06	0.01	0.28	0.59	0.30	2.42	13	0.07	0.03	0.86	0.33	0.01	1.45	1.73	0.37	4.43
AML with maturation (M2)	40	0.06	0.00	0.14	0.01	0.00	0.06	0.78	0.33	4.25	38	0.12	0.00	0.25	0.03	0.00	0.11	1.68	0.67	3.69
Others	11	0.00	0.00	0.05	0.01	0.00	0.07	1.04	0.29	9.90	12	0.37	0.02	0.92	0.21	0.00	16.50	1.22	0.29	31.80
**AML Classification (Prognostic)**																				
APL (M3)	17	0.06	0.01	0.11	0.15	0.00	0.68	3.23	0.34	15.70	14	0.06	0.03	0.20	0.02	0.00	0.13	0.71	0.29	3.94
All Others	61	0.04	0.00	0.13	0.01	0.00	0.07	0.75	0.32	2.56	58	0.11	0.01	0.39	0.04	0.00	0.30	1.49	0.51	3.45
**Myeloperoxidase Status**																				
Negative	13	0.05	0.02	0.09	0.15	0.03	0.67	3.82	0.63	21.90	12	0.10	0.01	0.24	0.03	0.00	0.65	1.36	0.75	4.93
Positive	58	0.05	0.00	0.13	0.01	0.00	0.08	0.66	0.31	2.38	55	0.09	0.01	0.30	0.04	0.00	0.26	1.22	0.30	3.39
**Sample Type**																				
Pre-chemotherapy Sample	32	0.04	0.00	0.08	0.04	0.00	0.31	1.33	0.30	2.42	31	0.10	0.01	0.59	0.04	0.01	0.44	1.05	0.21	3.24
Post-chemotherapy Sample	50	0.05	0.00	0.13	0.02	0.00	0.08	0.93	0.35	5.63	46	0.10	0.01	0.26	0.04	0.00	0.27	1.73	0.70	4.04
**Remission Status**																				
Resistant	32	0.05	0.00	0.14	0.01	0.00	0.09	0.70	0.25	2.35	31	0.12	0.01	0.30	0.05	0.00	0.33	1.48	0.44	3.98
Relapse	15	0.00	0.00	0.08	0.01	0.00	0.06	0.34	0.24	0.69	15	0.03	0.00	0.53	0.03	0.00	0.29	0.99	0.18	3.40
Persistent Remission	35	0.04	0.00	0.12	0.04	0.00	0.59	2.64	0.44	6.54	31	0.10	0.03	0.27	0.02	0.00	0.33	1.73	0.72	4.04
**Survival Status**																				
Dead	37	0.01	0.00	0.08	0.01	0.00	0.07	0.48	0.26	1.60	35	0.05	0.00	0.30	0.04	0.00	0.26	0.99	0.31	3.24
Alive	42	0.07	0.02	0.14	0.02	0.00	0.42	2.12	0.43	5.43	39	0.12	0.03	0.59	0.03	0.00	0.28	1.53	0.70	3.90

Results of Spearman’s correlation (*r*_s_) (Table 3) shows a moderate to strong significant positive correlation (*r*_s_ 0.6–0.94) between GRes and being alive, OS and DFS. There was a moderate to weak significant positive correlation (*r*_s_ 0.31–0.39) between marrow LRP expression and GRes, or being alive, whereas marrow MDR1 or MRP2 expression showed only very weak or no correlation with clinical outcomes. Blood MDR1, MRP2 and LRP showed only moderate to weak significant positive correlation with corresponding gene expression in marrow, but had no significant correlation with clinical outcomes.

**Table 3:** Spearman’s Correlation between various variables and gene expression in bone marrow and peripheral blood. Note that ‘M’ denotes Bone Marrow and ‘B’ denotes Peripheral Blood specimen.

Parameters		Persistant Remission	Survival Status (Post chemo)	Overall Survival (Weeks)	Disease Free Survival (Weeks)	Final Response	MDR1 express_Marrow (M)	MDR1 express_Blood (B)	MRP2 express_Marrow (M)	MRP2 express_Blood (B)	LRP express_Marrow (M)	LRP express_Blood (B)
Persistant Remission	Coefficient	1.000	0.672	−0.064	0.151	1.000	0.236	0.185	0.272	0.016	0.393	0.094
	p-value	.	<0.001	0.638	0.268	.	0.100	0.219	0.056	0.916	0.005	0.533
	N	56	53	56	56	56	50	46	50	46	50	46
Survival Status (Post chemotherapy)	Coefficient		1.000	0.315	0.327	0.600	0.258	0.187	0.116	−0.012	0.314	0.092
	p-value		.	0.003	0.017	<0.001	0.022	0.112	0.308	0.922	0.005	0.436
	N		86	86	53	86	79	74	79	74	79	74
Overall Survival (Weeks)	Coefficient			1.000	0.945	0.281	0.084	−0.056	0.107	0.202	0.169	−0.098
	p-value			.	<0.001	0.007	0.452	0.631	0.337	0.077	0.130	0.397
	N			90	56	90	82	77	82	77	82	77
Disease Free Survival (Weeks)	Coefficient				1.000	0.151	0.198	−0.116	0.212	0.222	0.275	−0.175
	p-value				.	0.268	0.167	0.443	0.139	0.139	0.054	0.245
	N				56	56	50	46	50	46	50	46
Final Response	Coefficient					1.000	0.068	0.075	0.241	−0.020	0.335	0.065
	p-value					.	0.545	0.518	0.029	0.863	0.002	0.575
	N					90	82	77	82	77	82	77
MDR1 Expression_Marrow (M)	Coefficient						1.000	0.324	0.110	0.138	0.157	0.153
	p-value						.	0.007	0.326	0.257	0.158	0.209
	N						82	69	82	69	82	69
MDR1 Expression_Blood (B)	Coefficient							1.000	−0.048	0.178	0.224	0.310
	p-value							.	0.696	0.122	0.064	0.006
	N							77	69	77	69	77
MRP2 Expression_Marrow (M)	Coefficient								1.000	0.507	0.375	−0.060
	p-value								.	<0.001	0.001	0.622
	N								82	69	82	69
MRP2 Expression_Blood (B)	Coefficient									1.000	0.018	0.003
	p-value									.	0.882	0.978
	N									77	69	77
LRP Expression_Marrow (M)	Coefficient										1.000	0.469
	p-value										.	<0.001
	N										82	69
LRP Expression_Blood (B)	Coefficient											1.000
	p-value											.
	N											77

Patient groups were compared as, (a) relapse or persistent remission, (b) GRes or PRes, (c) 1-year overall and disease-free survival (OS, DFS). Table 4 shows that marrow LRP expression is significantly higher in patients with persistent remission, being alive or GRes (p = 0.001, <0.001, 0.002 respectively). MDR1 or MRP2 expression was not significantly different. Interestingly, marrow LRP expression was significantly higher among known favorable prognostic factors, i.e., acute promyelocytic leukemia (APL; M3), and negative MPO. Patients with low marrow LRP expression were 10 times more likely to end up with relapse, 6 times more likely to die within one year and 4.4 times more likely to end up as PRes as compared to patients with high marrow LRP.

**Table 4:** Chi-square analysis and Odds ratios between various variables. All df = 1.

Parameters	Groups	N	χ2 Value	p-value	Odds Ratio	95% CI	
						Lower	Upper
**AML Classification (APL vs. All others)**							
Gender	Male	61	0.159	0.690	1.286	0.372	4.446
	Female	23					
MPO	Negative	12	13.692	<0.001	11.200	2.614	47.992
	Positive	61					
FLT3	Negative	32	0.008	1.000	1.111	0.109	11.330
	Positive	7					
Karyotyping	Unfavorable	17	2.378	0.165	0.281	0.053	1.503
	Favorable	28					
Remission Status	Relapse	16	6.320	0.012	0.095	0.011	0.807
	Persistent Remission	34					
Survival Status	Dead	39	4.279	0.052	0.286	0.083	0.980
	Alive	42					
Final Response	Poor	50	15.513	<0.001	0.091	0.024	0.353
	Good	34					
**Persistent Remission (Relapse vs. Persistent Remission**							
Gender	Male	45	0.036	1.000	0.875	0.221	3.464
	Female	11					
AML Classification	APL (M3)	15	6.320	0.019	0.095	0.011	0.807
	Others	35					
MPO Status	Negative	11	5.184	0.033	0.112	0.013	0.966
	Positive	36					
FLT3	Negative	26			invalid		
	Positive	—					
Karyotyping	Unfavorable	11	1.239	0.450	0.413	0.085	2.001
	Favorable	21					
**Survival Status (Deard vs. Alive)**							
Gender	Male	63	1.819	0.177	0.514	0.194	1.362
	Female	23					
AML Classification	APL (M3)	16	4.279	0.052	0.286	0.083	0.980
	Others	65					
MPO Status	Negative	13	1.049	0.306	0.530	0.156	1.806
	Positive	61					
FLT3	Negative	33	1.558	0.407	0.333	0.056	1.971
	Positive	7					
Karyotyping	Unfavorable	17	0.061	0.805	0.860	0.260	2.843
	Favorable	30					
Remission Status	Relapse	19	23.922	<0.001	28.125	6.162	128.360
	Persistent Remission	34					
Final Response	Poor	52	30.929	<0.001	20.357	6.071	68.262
	Good	34					
**Final Response (Poor vs. Good)**							
Gender	Male	66	1.929	0.165	0.494	0.181	1.350
	Female	24					
AML Classification	APL (M3)	17	15.513	<0.001	0.091	0.024	0.353
	Others	67					
MPO Status	Negative	14	8.050	0.007	0.177	0.049	0.635
	Positive	62					
FLT3	Negative	35	5.169	0.033	invalid		
	Positive	7					
Karyotyping	Unfavorable	18	0.385	0.535	0.688	0.210	2.250
	Favorable	31					
Survival Status	Dead	42	30.929	<0.001	20.357	6.071	68.262
	Alive	44					
**Gene Expression:**							
**Remission Status (Relapse vs Persistent Remission)**							
MDR1 expression - Marrow	Low (<1)	47	1.368	0.545	invalid		
	High (≥1)	3					
MRP2 expression - Marrow	Low (<1)	46	0.052	1.000	1.313	0.125	13.744
	High (≥1)	4					
LRP expression - Marrow	Low (<1)	22	11.271	0.001	10.000	2.317	43.160
	High (≥1)	28					
MDR1 expression - Blood	Low (<1)	40	0.002	1.000	0.963	0.156	5.954
	High (≥1)	6					
MRP2 expression - Blood	Low (<1)	41	0.406	1.000	2.074	0.211	20.367
	High (≥1)	5					
LRP expression - Blood	Low (<1)	19	1.328	0.249	2.078	0.593	7.275
	High (≥1)	27					
**Survival Status (Dead vs. Alive)**							
MDR expression - Marrow	Low (<1)	73	0.475	0.679	1.842	0.317	10.690
	High (≥1)	6					
MRP expression - Marrow	Low (<1)	74	1.544	0.364	3.789	0.404	35.532
	High (≥1)	5					
LRP expression - Marrow	Low (<1)	40	13.896	<0.001	6.023	2.267	15.999
	High (≥1)	39					
MDR expression - Blood	Low (<1)	64	0.247	0.740	1.409	0.363	5.473
	High (≥1)	10					
MRP expression - Blood	Low (<1)	68	0.019	1.000	0.889	0.167	4.720
	High (≥1)	6					
LRP expression - Blood	Low (<1)	31	2.481	0.115	2.118	0.828	5.418
	High (≥1)	43					
**Final Response (Poor vs. Good)**							
MDR expression - Marrow	Low (<1)	76	0.142	1.000	1.375	0.260	7.259
	High (≥1)	6					
MRP expression - Marrow	Low (<1)	77	0.653	0.646	2.109	0.333	13.358
	High (≥1)	5					
LRP expression - Marrow	Low (<1)	40	9.981	0.002	4.412	1.716	11.343
	High (≥1)	42					
MDR expression - Blood	Low (<1)	67	0.000	1.000	0.988	0.254	3.833
	High (≥1)	10					
MRP expression - Blood	Low (<1)	69	0.352	0.707	1.556	0.358	6.751
	High (≥1)	8					
LRP expression - Blood	Low (<1)	31	0.492	0.483	1.399	0.547	3.576
	High (≥1)	46					

Binary logistic regression analysis was conducted to predict therapeutic outcome (PRes vs GRes) (Table 5). A test of the full model against a constant-only model was statistically significant, indicating that the predictors as a set reliably distinguished between PRes and GRes (58.3% vs 68.3%; χ² (df = 8, N = 90) = 19.5, p = 0.013; Hosmer-Lemeshow significance = 0.15). Nagelkerke’s R^2^ of 0.37 indicated a moderate relationship between prediction and grouping. Prediction success overall was 68.3% (65.7% for PRes and 72% for GRes). The Wald criterion demonstrated that a diagnosis of APL and LRP expression in marrow made a significant contribution to the prediction of GRes.

**Table 5:** Logistic Regression Analysis of Study Model to predict Therapeutic outcome (poor vs good responders).

Parameters	B	S.E.	Wald	df	p	Exp(B)	95% CI	
							Lower	Upper
N = 60; Nagelkerke’s R2 = 0.37; χ2(8) = 19.45, p < 0.013 (For *Good* Response)								
AML Class (APL/Others)	2.427	1.070	5.143	1	0.023	11.328	1.390	92.303
MPO	0.578	0.921	0.394	1	0.530	1.783	0.293	10.838
***Bone Marrow (Gene expression, low vs. high)***								
-MDR1	2.133	1.490	2.051	1	0.152	8.443	0.456	156.465
-MRP2	−1.412	1.519	0.864	1	0.353	0.244	0.012	4.783
-LRP	−1.843	0.771	5.708	1	0.017	0.158	0.035	0.718
***Peripheral Blood (Gene expression, low vs. high)***								
-MDR1	−0.152	1.167	0.017	1	0.897	0.859	0.087	8.460
-MRP2	−1.276	1.324	0.930	1	0.335	0.279	0.021	3.734
-LRP	−0.095	0.829	0.013	1	0.908	0.909	0.179	4.619
Constant	0.743	2.114	0.123	1	0.725	2.101		

**Figure 1:**
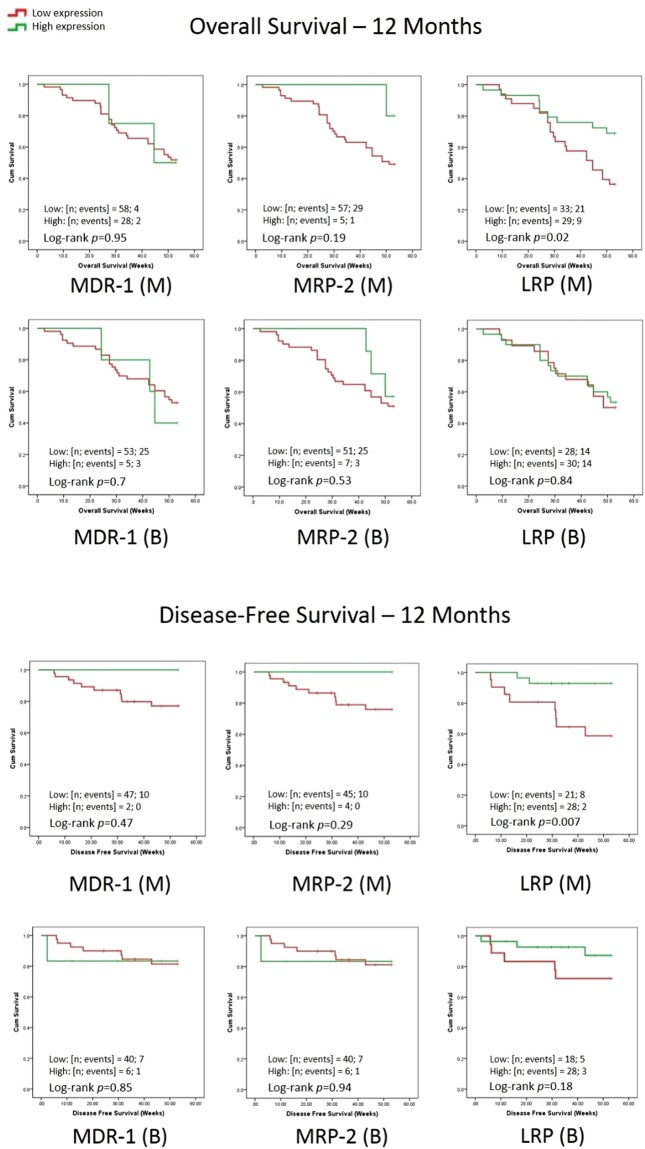
Kaplan-Meier Survival Analysis of AML patients in relation to MDR-1, MRP-2 and LRP gene expression. Note the overall as well as disease-free survival over 12 months.

Kaplan-Meier analysis for 1-year DFS and OS showed that MDR1 and MRP2 expression did not have any significant effect. However high marrow LRP expression was significantly associated with better OS (p = 0.02) and DFS (p = 0.007) (Fig. 1).

## Discussion

In this study we observed a high marrow LRP expression predicting reduced relapse rate and better 1-year DFS and OS. A diagnosis of APL was another favorable predictor, in agreement with the scientific literature. Although, expression of LRP correlated positively in bone marrow and peripheral blood, the results of blood samples did not correlate with clinical outcome, thus suggesting a possible differential role of tissue-specific gene expression in this regard. Patients with low marrow LRP responded poorly, relapsed and had less survival likelihood than those with high expression. Neither MDR1 nor MRP2 expression in marrow or blood could predict remission, relapse, and 1-year DFS or OS. The strengths of our study include inclusion of a single type of disease and treatment protocol, utilization of both bone marrow and peripheral blood separately without pooling them together, prospective follow up of the patients, and a sample size larger than many other such studies. Being a single-center study is a limitation of our study. Please see Supplementary Table 1 for a summary of scientific evidence discussed in this section.

### MDR1 and AML Therapeutic Outcome

Several studies have reported MDR1 expression in association with therapeutic outcome in various cancers. In agreement with our findings some studies reported no effect of MDR1 expression on clinical outcome in AML patients treated with different anticancer drugs (n = 30)^18^, or in a non-homogenous group of acute leukemias (AML + ALL), although an inverse relationship with 2-year OS was noted in acute leukemias (n = 71)^10^.

However, some studies with a larger number of AML patients (n = 211, 331) have related MDR1 overexpression with a lower CR rate^5,6^, albeit using a heterogenous patient population, different treatment protocols and less sensitive techniques such as semi-quantitative RT-PCR or flowcytometry. No effect on DFS or OS was observed by one of those studies despite better CR among those who had lower MDR1 expression as well as favorable cytogenetic markers (and vice versa)^6^, while the other study reported no effect of MDR1 expression among the subpopulation (n = 123/331) who were treated like patients in our study^5^. Interestingly, some studies with a sample size lower than ours but on a different drug protocol have shown that MDR1 overexpression correlated with lower CR and higher relapse rates in acute leukemia (AL) (n = 44)^7^ and with reduced DFS in acute lymphoblastic leukemia (ALL) patients treated with ALL-BFM 95 protocol (n = 49)^19^. Thus, a clear association observed in a real clinical situation needs further evidence.

Studies on solid tumors treated with chemotherapy protocols different than those for AML or ALL patients, have also exhibited conflicting results. In an ovarian cancer study (n = 61) MDR1 overexpression was found associated with reduced progression free survival (PFS) and OS but not with chemotherapy response^8^. A study on breast cancer (n = 59) reported MDR1 overexpression in patients with decreased response and PFS^16^. Another study on breast cancer patients (n = 220) reported undetectable or very low MDR1 by immunohistochemistry and RT-PCR^20^. Yet another study reported no association of MDR1 overexpression with a clinical outcome in breast cancer tissue (n = 54) compared to normal breast tissue^21^.

One *in vitro* study has reported changes in MDR1 expression after exposure to cytarabine in both drug-resistant and sensitive leukemic cells, but this could not be related to a change in clinical outcome for obvious reasons^22^. Similarly, another study conducted on breast cancer cell lines as well as breast cancer specimens (n = 168), demonstrated no significant change in MDR1 expression after anthracycline chemotherapy^20^. In our study we observed that patients with ‘AML without maturation’ had higher MDR1 expression in marrow as compared to ‘AML with maturation’. In a previous study on 13 different cell lines it was observed that MDR1 was overexpressed in CD34^+^ AML cells compared to CD34^−^ cells^23^. Thus, it appears that MDR1 may be associated with a specific subset of AML patients, which partly explains the conflicting results in the scientific literature. Recently, research has focused on finding an effective MDR1 inhibitor^24,25^. However without a clear understanding of the role of MDR1, it may not achieve better clinical results.

### MRP2 and AML Therapeutic Outcome

MRP2 is also implicated to drug resistance in hematological as well as solid tumors, although with conflicting results similar to those described above for MDR1. MRP2 overexpression is associated with relapse in AML patients (n = 30)^18^ and with lower 2-year survival in acute leukemias (n = 71)^10^ with reduced RFS in ALL patients (n = 105)^26^, as well as with poor response to chemotherapy comprising of 5-flurouracil, doxorubicin and cisplatin in esophageal squamous cell carcinoma^13^. Some *in vitro* studies have demonstrated a correlation between overexpression of MRP2 and resistance to antineoplastic drugs^8,12^. Normally, MRP2 expression on hepatocytes is much greater than in other tissues. A study of rat hepatocytes showed that MRP2 negative cells showed high sensitivity when treated with cisplatin due to high intracellular platinum accumulation, but when tested in ovarian cancer patients, they did not find this effect^27^. Similarly, some other studies also could not find any association of MRP2 with chemotherapy outcome, such as in breast cancer patients (n = 59) treated with either anthracyclines or hormone therapy or both^16^, or in ovarian carcinoma patients (n = 61)^8^ treated with different protocols that included platinum-containing drugs. Our results are in agreement with such studies as we found no association between MRP2 expression and any therapeutic outcome. Hence it could be possible that MRP2 may play a role in drug efflux and thereby in drug resistance in a tissue specific manner, such as liver, but not in AML.

### LRP and AML Therapeutic Outcome

As described earlier, LRP and vaults play an important role in nucleocytoplasmic transport, apoptosis, DNA damage repair, cellular detoxification and chemotherapy resistance^28,29^. Some animal and *in vitro* studies reported no association of LRP expression with resistance to cytotoxic drugs^30,31^. However, Mashima *et al*.^32^ suggested that doxorubicin can bind vRNA which can then be transported by vaults between cytoplasm and nucleus. Another *in vitro* study suggests that LRP transports doxorubicin out of nucleus, resulting in the observed resistance to apoptosis following doxorubicin treatment and is reversed by *in vitro* inhibition of LRP, vPARP and TEP1^33^. As described earlier, vaults have MVP, vPARP, TEP1 and vRNA as part of their structure. TEP1 forms telomeres and thus prevent cancer formation. Interestingly, we found significant differences in bone marrow but not in peripheral blood samples, which might be suggestive of a role of LRP in combating the carcinogenesis at the initial stage of disease development, especially in hematopoietic stem cells. In fact, it has been postulated that premature aging in normal hematopoietic stem cells induced by chemotherapy or ionizing radiation may result in growth advantage for malignant cells^34^. The aging is minimized by telomerase activity, and thus increased MVP expression may favor growth of normal bone marrow. However, only clinical studies have the potential to prove its implication in terms of therapeutic response. Some studies reported no association of LRP expression with chemotherapy outcome in AML patients (n = 331, 352)^5,6^ or ALL patients (n = 49, n = 27)^19,35^. However, patients studied by Schaich *et al*.^5^ received double induction chemotherapy with higher dose of daunorubicin (60 mg/kg/m2/d) as compared to patients in our study (45 mg/kg/m2/d). Such differences in chemotherapy doses could influence the outcome as described by Afsar *et al*.^36^.

On the other hand, several studies point towards the role of LRP in adverse therapeutic outcomes. Positive LRP expression correlated with lower CR rate but not with relapse rate in acute leukemias^10^. It also correlated with poor response and prognosis and lower OS in testicular tumor (n = 70)^17^, and lung cancer (n = 92)^37^. LRP overexpression is associated with reduced CR rate in AML patients (n = 67)^38^, decreased DFS in pediatric ALL patients (n = 30)^9^, and poor prognosis in breast cancer patients (n = 59)^16^. However, results of many such studies should be regarded with caution due to different sample sizes, different analysis methods, or differences in tumor biology or treatment.

Our results disagree with many studies described above. Hence, we explored online OncoLnc® database (http://www.oncolnc.org/search_results/?q = mvp) for further evidence about LRP (MVP). The database-generated Kaplan-Meier curves showed that in invasive carcinoma of breast (denoted as BRCA) and renal papillary cell carcinoma (denoted as KIRP), higher LRP or MVP expression is associated with significantly better survival, thus agreeing with our results. Sarcoma (denoted as SARC) also showed significantly better survival among high LRP expressors, but only when the first and last quartiles were considered. The Cox coefficients for all three diseases (*BRCA*: −0.23; *KIRP*: −0.37; *SARC*: −0.34; all *p*-values < 0.05) also supported such findings, but their adjusted p-values (q-values) failed to reach statistical significance. The database also shows that in AML (denoted as LAML; comprised of a mixed patient population, with a lower sample size) a high LRP expression is associated with poor survival, however statistical significance was not achieved unless at least the top and bottom one third of gene expression values were considered while constructing the survival curve online. The survival curves are given as Supplementary Fig. 2. As LRP is a part of vault structure, the role of LRP as a favorable predictor in AML chemotherapy can be explained on the basis that LRP (and vaults) may be involved in transporting anticancer drugs inside the nucleus. However, further studies are needed to verify this hypothesis.

To conclude, in AML patients treated with standard dose 3 + 7 cytarabine and daunorubicin regimen, MDR1 and MRP2 gene expression in bone marrow and peripheral blood samples have no association with remission, resistance or relapse, nor with 1-year DFS or OS. However, higher bone marrow expression of LRP predicts better CR rate, persistent remission and 1-year DFS and OS. Additionally, our model of logistic regression endorses LRP and APL as significant predictors for a good chemotherapeutic response. To the best of our knowledge, our results are the first to show that LRP expression is a predictor of favorable outcome in a commonly used AML chemotherapy.

Further research is warranted to explore the mechanism and regulation of LRP expression, and its interaction with other molecular pathways. Studies are also needed to evaluate the role of LRP as a predictor in different cancers and chemotherapy protocols. We also recommend that further studies with a larger sample size and better techniques should be conducted to clarify the role of xenobiotic transporters in chemotherapy resistance and clinical outcomes.

## Methods

We recruited 135 AML patients, newly diagnosed according to WHO criteria and treated at National institute of Blood Diseases and Bone Marrow Transplantation (NIBD&BMT), Karachi, during 2011–2017. All prospective AML patients, including acute promyelocytic leukemia (APL) patients, were included if they received an induction chemotherapy comprising only of the standard 3 + 7 regimen (daunorubicin 45 mg/m^2^ on days 1–3; cytarabine 200 mg/m^2^ on days 1–7). Bone marrow (BM) and blood samples of patients were collected separately. 45 patients were excluded for other reasons, such as hemolyzed samples or no RNA yield. Thus, a total of 90 AML patients were included. Sample collection, storage, enrichment, RNA extraction and reverse transcription reaction were carried out as described previously^39^. The study was approved by the Ethical Review Board at NIBD&BMT in accordance with the Declaration of Helsinki. A written informed consent to participate in this research was given by all patients, or by legal guardians if the patient was below the age of 18-years.

Chemotherapy response, which included complete remission (CR) after first induction chemotherapy, resistance, relapse, overall survival (OS), and disease-free survival (DFS), was defined as described by Döhner *et al*.^3^.

### Real-Time/Quantitative Polymerase Chain Reaction (qPCR)

We used Eco Illumina System version 5.0.16.0 (Illumina, CA, USA). A commercially available VeriQuest Probe qPCR Master Mix (Affymerix, CA, USA) was used. Glyceraldehyde 3-phosphate dehydrogenase (GAPDH) gene expression remained internal control in the experiments. Primers and probes were purchased from Integrated DNA Technologies (IDT, IA, USA). The reporter dye in the probe was 6-carboxyfluorescein (FAM) and the quencher was 6-carboxytetramethylrhodamine (TAMRA) with an intermediate ZEN-BQI. The primers and probes used for MDR1 were: forward 5′-GGAAGCCAATGCCTATGACTTTA-3′, reverse 5′-GAACCACTGCTTCGCTTTCTG-3′, probe 5′-/56-FAM/TGAAACTGC/ZEN/CTCATAAATTTGACACCCTGG/3IABkFQ/-3′; for MRP2 were: forward 5′-ATGCTTCCTGGGGATAAT-3′, reverse 5′-TCAAAGGCACGGATAACT-3′, probe 5′-/56-FAM/TGTATCTGT/ZEN/TCAGATGTTTTATGTGTCTACCT/3IABkFQ/-3′; for LRP were: forward 5′-CAGCTGGCCATCGAGATCA-3′, reverse 5′-TCCAGTCTCTGAGCCTCATGC-3′, probe 5′-/56-FAM/CAACTCCCA/ZEN/GGAAGCGGCGGC/3IABkFQ/−3′, and for GAPDH were: forward 5′-GAAGGTGAAGGTCGGAGTCA-3′, reverse 5′-GAAGATGGTGATGGGATTTC-3′, probe 5′-(FAM)/56-JOEN/CCGACTCTT/ZEN/GCCCTTCGAAC/3IABkFQ/(TAMRA)-3′^16,40^. The reaction conditions and details were described previously^39^.

### Statistical Analysis

Data was analyzed using SPSS ver. 19.0 software. Qualitative variables were given as frequency and percentage while quantitative variables were described using medians and interquartile ranges where appropriate. Gene expression was calculated from assay Cq values normalized to healthy control blood samples using 2^−ΔΔCt ^^41^.

As the gene expression data was not normally distributed, patients with gene expression <1 were categorized as low expressers, while those with gene expression >1 were categorized as high expressers. For non-parametric variables, Chi-square test of independence or Fisher Exact test was carried out, and odds ratios were computed where appropriate. Spearman’s correlation was computed between gene expression and clinical outcome. Binary logistic regression analysis was carried out to estimate the predictive value of our model. Kaplan-Meier analysis (log-rank test) was used to estimate 1-year OS and DFS. Only a p-value < 0.05 was considered significant.

## Electronic supplementary material

Supplementary table and figures
