# Supplementary material for: Lung resistance-related protein (LRP) predicts favorable therapeutic outcome in Acute Myeloid Leukemia
Source: Sci Rep. 2019 Jan 23;9:378. doi: 10.1038/s41598-018-36780-8 (PMC6344578; doi:10.1038/s41598-018-36780-8)

Lung resistance-related protein (LRP) predicts favorable therapeutic outcome in Acute Myeloid Leukemia

Running Title: LRP predicts favorable AML outcome

Authors:

*Bibi Kulsoom,[1,2] Tahir Sultan Shamsi[1] and Nasir Ali Afsar[2]

[1]National Institute of Blood Diseases and Bone Marrow Transplantation, Karachi, Pakistan.

[2]Jinnah Medical and Dental College, Karachi, Pakistan.

Correspondence:

*Bibi Kulsoom. *Email* drknpk@yahoo.com . *Mailing Address*: National Institute of Blood Diseases and Bone Marrow Transplantation, Karachi, Pakistan. *Phone* +92 332 993 6614.

Supplementary Table 1: Summary of therapeutic outcomes in relationship with MDR1, MRP2 and LRP. Note the citations as given in the 'References' section in the article.

Cancer (n)	N [citation]	Drugs	Techni-ques	*Effect of INCREASED gene expression on:*			
				CR	Relapse	DFS	OS
**Multidrug resistance protein 1 (MDR1)**							
AML	331 [5]	Daunorubicin, Cytarabine	SqPCR	Decreased	-	-	Decreased
	211 [6]		FCM	Decreased	-	None	None
	30 [18]	Daunorubicin, Idarubicin, Etoposide, Mitoxantrone	qPCR, FCM	-	None	-	-
AML, ALL	26, 18 [7]	Anthracyclines, Vincristine, Etoposide	qPCR	None	Increased	-	-
	39, 32 [10]	AML: Cytarabine, Idarubicin,<br>ALL: Vincristine, Prednisone, Daunorubicin, L-asparaginase	sqPCR	None	None	-	Decreased
ALL	49 [19]	BFM95 Protocol: Methotrexate, Vincristine, Prednisone, Daunorubicin, L-asparaginase	sqPCR	-	Increased	Decreased	-
	30 [9]	Dexametasone, Vincristin,<br>Daunomycin, TIT (Methotrexate, Cytarabine, Dexamethasone), L-asparaginase, Cytarabine	sqPCR	None	-	None	None
Breast	59 [16]	FAC/FEC	qPCR	Decreased	-	Decreased	-
	54 [21]	FAC/FEC	qPCR	None	None	None	None
Ovary	61 [8]	Carboplatin, Paclitaxel, Cyclophosphamide, Treosulfan, Topotecan, Gemcitabine	sqPCR	None	-	Decreased	Decreased
**Multidrug resistance associated protein 2 (MRP2)**							
AML	30 [18]	Daunorubicin, Idarubicin, Etoposide, Mitoxantrone	qPCR, FCM	-	None	-	-
ALL	105 [26]	Vincristine, Dexamethasone, L-asparaginase	qPCR	-	Increased	Decreased	-
AML, ALL	39, 32 [10]	AML: Cytarabine, Idarubicin<br>ALL: Vincristine, Prednisone, Daunorubicin, L-asparaginase	sqPCR	-	-	-	Decreased

Breast	59 [16]	FAC/FEC	qPCR	None	-	-	-
Ovary	61 [8]	Carboplatin, Paclitaxel, Cyclophosphamide, Treosulfan, Topotecan / Gemcitabine	sqPCR	None	-	Decreased	-
Ovary + in vitro Hepatocyte	22 [27] + in vitro	Cisplatin	IHC	low drug sensitivity	-	-	Increased (patients)
Esophagus	81 [13]	Cisplatin	IHC, qPCR	-	-	-	Decreased
**Lung resistance related Protein (LRP) or major vault protein (MVP)**							
AML	331 [5], 211 [6]	Daunorubicin, Cytarabine	sqPCR	None	-	-	-
	67 [38]		sqPCR	Decreased	-	-	-
AML, ALL	39, 32 [10]	AML: Cytarabine, Idarubicin,<br>ALL: Vincristine, Prednisone, Daunorubicin, L-asparaginase	sqPCR	Decreased	-	-	-
ALL	30 [9]	Dexametasone, Vincristin, Daunomycin, TIT (Methotrexate, Cytarabine, Dexamethasone), L-asparaginase, Cytarabine	sqPCR	None	Increased	Decreased	Decreased
	49 [19]	BFM95 Protocol: Methotrexate, Vincristine, Prednisone, Daunorubicin, L-asparaginase	qPCR	None	-	-	-
	45 [35]	Dexamethasone, 6-mercaptopurine, L-asparaginase	qPCR	Decreased	-	-	-
NSCLC	92 [37]	Vinorelbine, Cisplatin	IHC	-	-	-	Decreased
Testis	70 [17]	Cisplatin	IHC, WB	Decreased	-	-	Decreased
Breast	59 [16]	FAC/FEC	qPCR	-	-	Decreased	-

Abbreviations:

ALL: Acute lymphoblastic leukemia; **AML**: Acute myeloid leukemia; **CR**: Complete remission; **DFS**: Disease-free survival; **FAC/FEC**: fluorouracil, doxorubicin/ epirubicin, cyclophosphamide; **FCM**: Flow cytometry; **IHC**: Immunohistochemistry; **NSCLC**: Non-small cell lung cancer; **OS**: Overall survival; **qPCR**: Quantitative polymerase chain reaction; **sqPCR**: Semi-quantitative polymerase chain reaction; **WB**: Western blotting.

Supplementary Figure 1: Boxplots showing gene expression (logarithmic scale) of MDR-1, MRP-2, and LRP in bone marrow (M) and peripheral blood (B). Note the uniform distribution (linear scale) of the Cq values of the house-keeping gene GAPDH in bone marrow and blood samples.

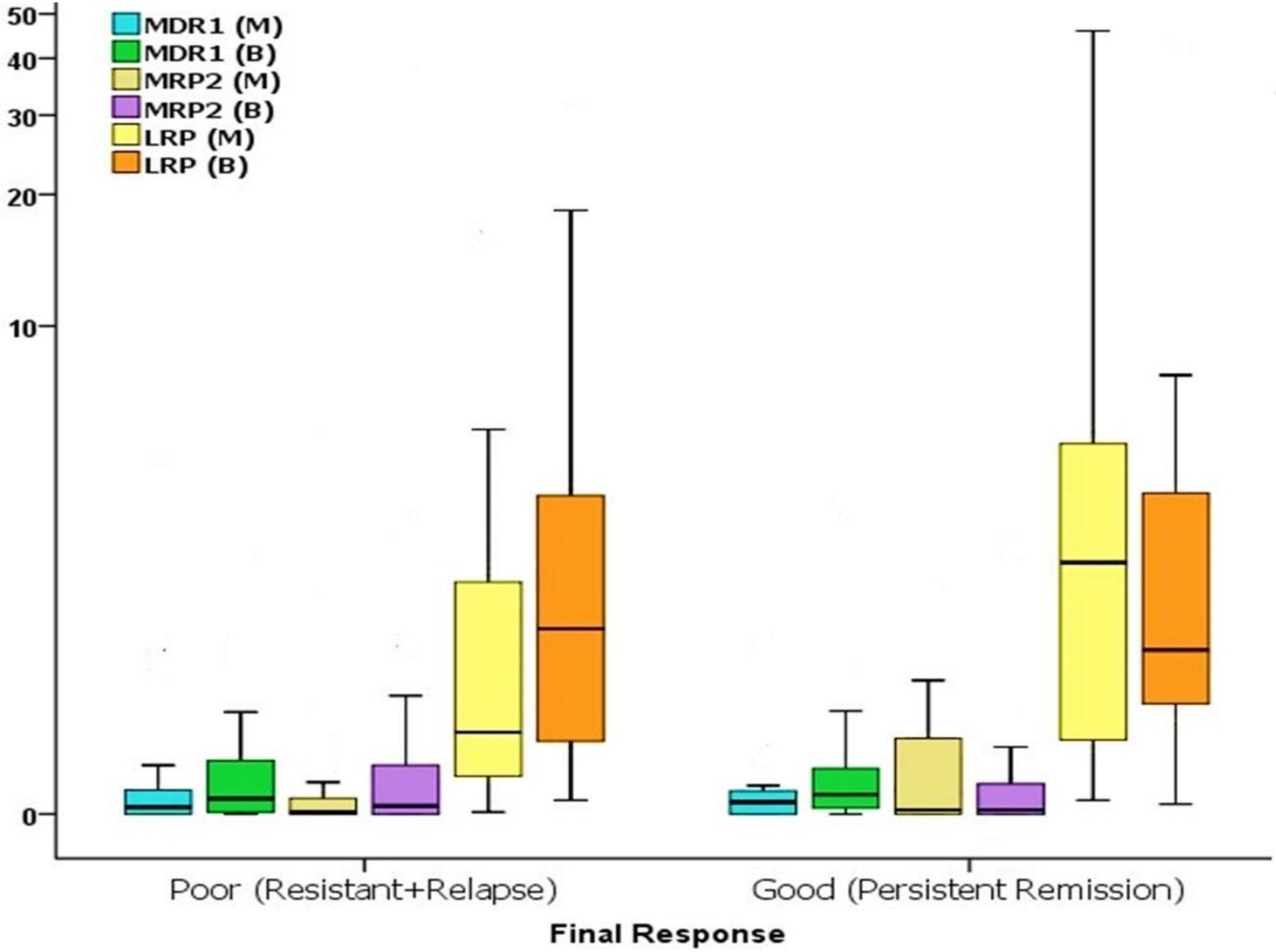

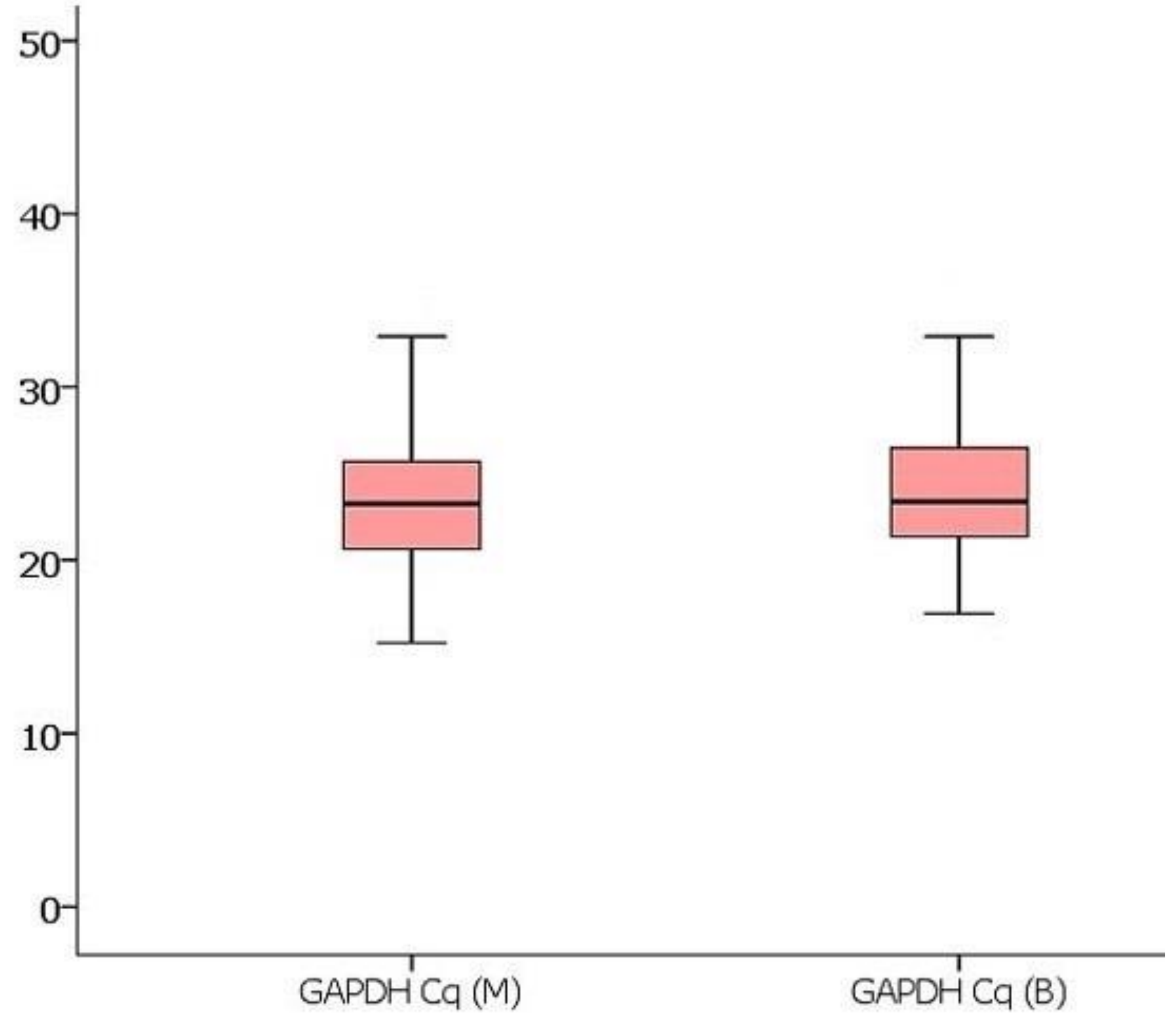

Supplementary Figure 2: Kaplan-Meier survival curves at OncoLnc® database (www.oncolnc.org/search_results/?q=mvp) according to LRP (MVP) expression in invasive carcinoma of breast (BRCA), renal papillary cell carcinoma (KIRP), sarcoma (SARC), and AML (LAML). The adjusted p-values (q-values) are given.

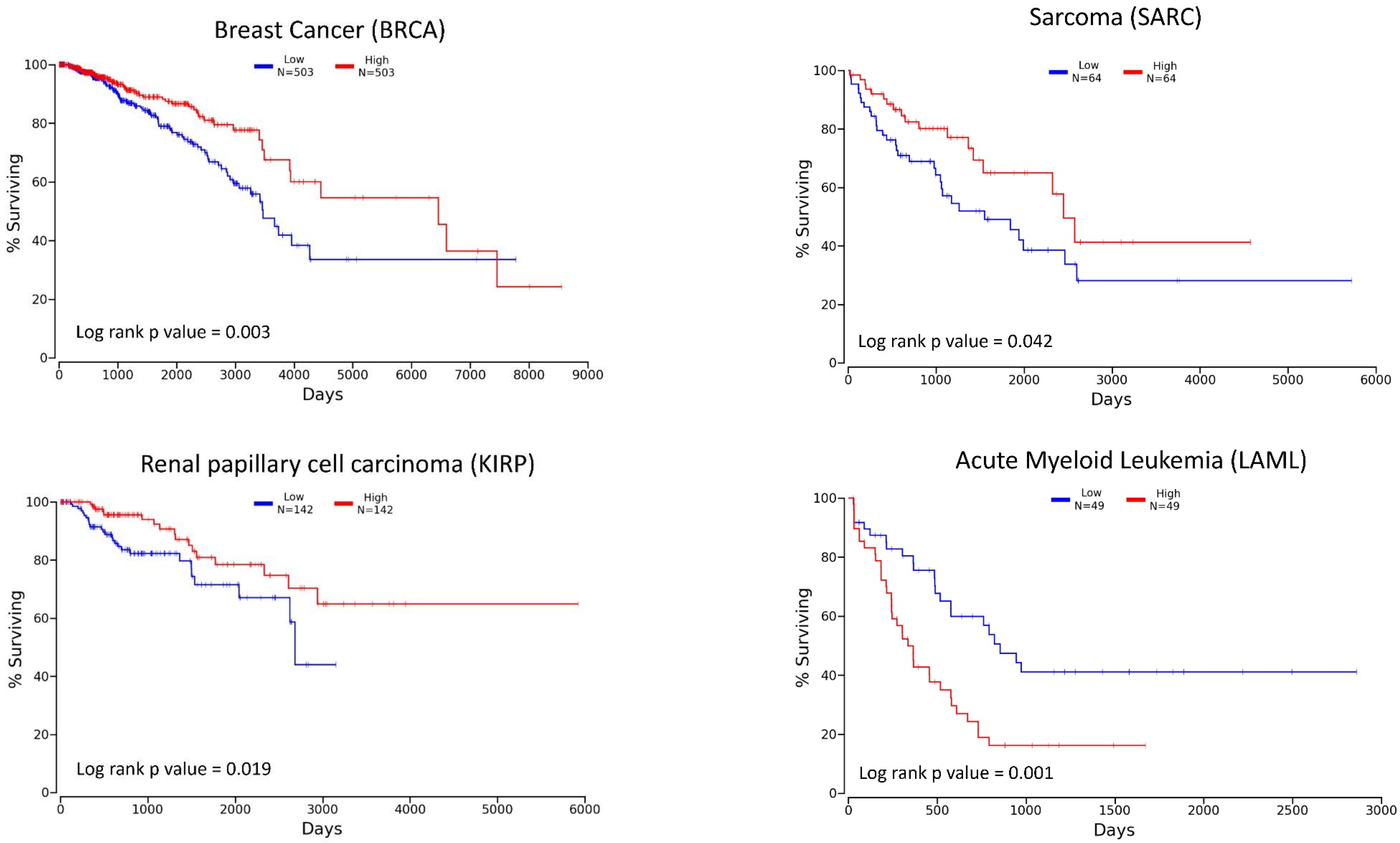

Supplement: Supplementary file 1 — Supplementary table and figures [file 41598_2018_36780_MOESM1_ESM.pdf]
